# Supplementary material for: The α5-α6-α7-Pba3-Pba4 Complex: A Starting Unit in Proteasome Core Particle Assembly
Source: Biomolecules. 2025 May 8;15(5):683. doi: 10.3390/biom15050683 (PMC12109424; doi:10.3390/biom15050683)
Supplement: Supplementary file 1 [file biomolecules-15-00683-s001.zip › biomolecules-3592808-Figures S1-S8.pdf]

Matias, A.C., Tiago, M.N., Zimmermann, J., Dohmen, R.J. & Ramos, P.C.

Supplementary Figures (S1-S8)

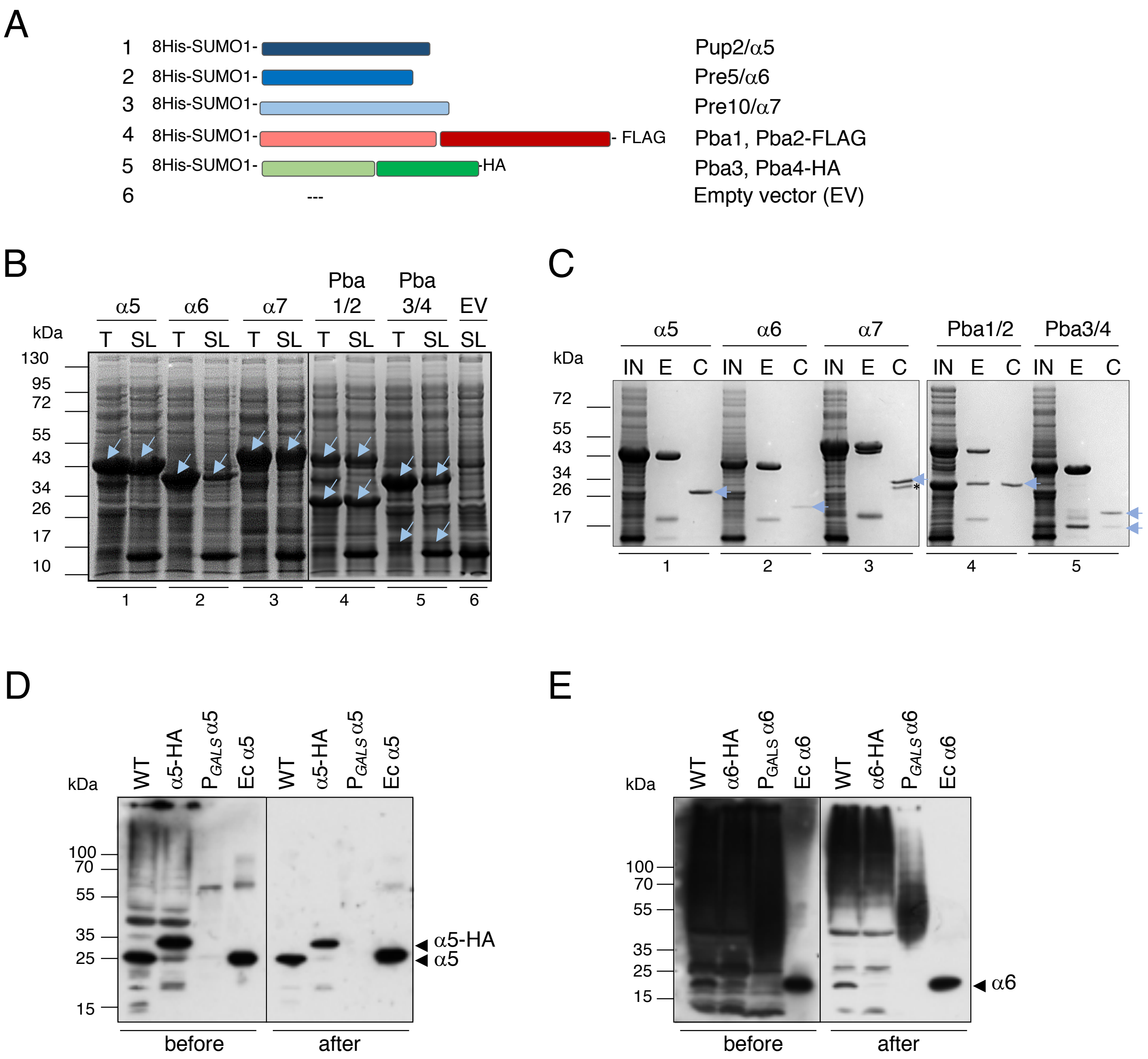

Figure S1. **Expression and purification of α-subunits and Pba chaperones, antibody validation.** **(A)** Schematic representation of produced proteasome subunits Pup2/α5 (plasmid pJZ20), Pre5/α6 (plasmid pJZ21), Pre10/α7 (plasmid pJZ22) and the chaperone pairs Pba1-Pba2 (plasmid pJZ24) and Pba3-Pba4 (plasmid pJZ23). The proteasome subunits as well as Pba1 and Pba3 were fused to 8His-SUMO1. Expressed from operons together with the latter two polypeptides, Pba2 contains additionally a FLAG epitope, and Pba4 an HA tag at the C-terminus. **(B)** Comparison of total boiled extracts (T) and soluble (SL) extracts produced by sonication of *E. coli* cells expressing the indicated proteins after 12 % SDS-PAGE followed by Coomassie-staining. The empty vector (EV) serves as control. Recombinantly expressed proteins are indicated by arrows. **(C)** The indicated proteasome subunits and chaperones were purified using consecutive Ni^{2+}-NTA chromatography. The 8His-SUMO1 tag was removed during the procedure using SENP1. Samples of the input (IN), material eluted from Ni-NTA resin ("E"), and after SUMO1 cleavage ("C") was analyzed by 12 % SDS-PAGE and Coomassie staining. **(D)** and **(E)**, validation of recombinantly expressed α-subunits and antibody specificity before and after removal of non-specific antibodies as described in Materials and Methods section 2.5. Yeast extract from wild-type (WT) yeast cells, or from cells expressing α5 or α6, with or without HA tag, or the respective purified subunit recombinantly produced in *E. coli* (Ec). In addition, yeast strains expressing either α5 or α6 subunits under control of the galactose-inducible promoter P_{GALS} (see Table 1) were used. After cells were grown in galactose, they were shifted to glucose media to repress synthesis of the α5 and α6 proteins. Absence of the signals for the respective α-subunits in these extracts confirms their specific recognition in the other extracts.

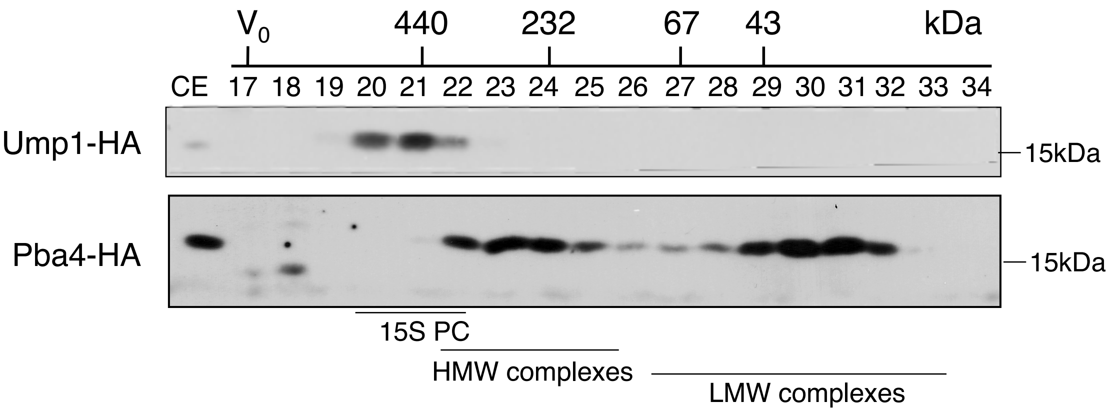

Figure S2. Pba3-Pba4 containing complexes are distinct from 15S-PC. Gel filtration from WT cells crude extracts expressing either Ump1-HA or Pba4-HA in exactly the same conditions. The bottom panel is the same as in Fig.1A for comparison.

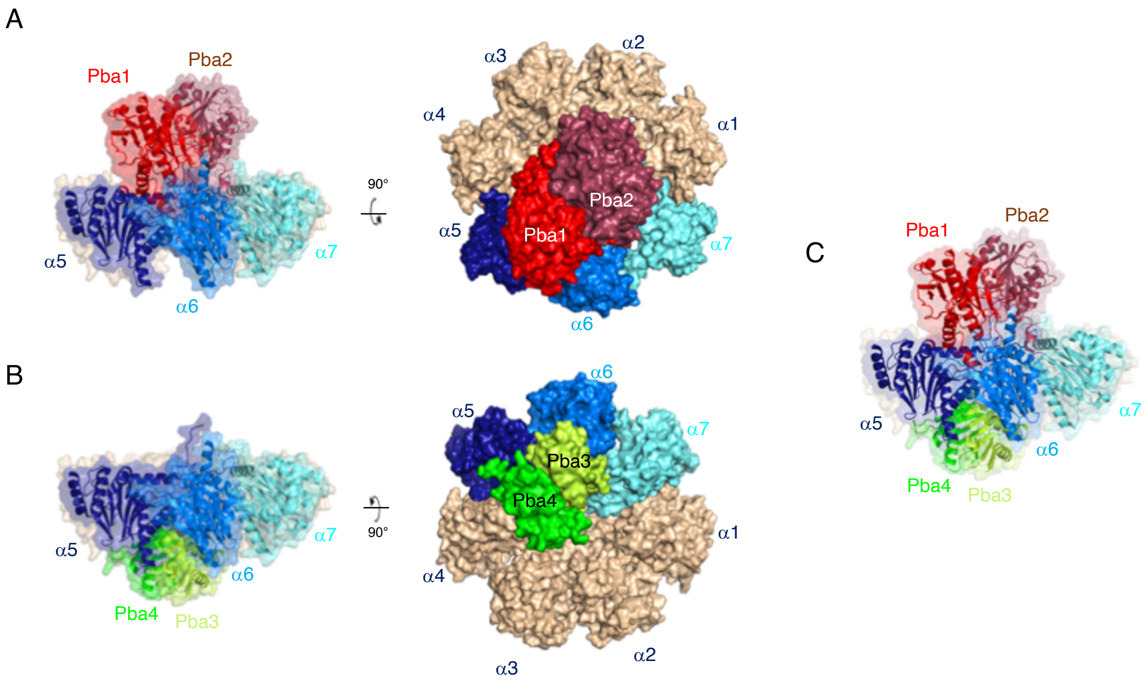

Figure S3. Modelling of the chaperones Pba1/Pba2 and Pba3/Pba4 on a complete proteasome α-ring. **(A)** Position of the chaperone Pba1-Pba2 on the outer surface of the α-ring, late-PC (PDB: 8RVL [18]), indicated are the subunits with extensive contact. **(B)** Modelling of the chaperone Pba3-Pba4-α5 unit (PDB: 2Z5C [9]) onto the late-PC. **(C)** Hypothetical complex of both chaperone pairs and the indicated α-subunits.

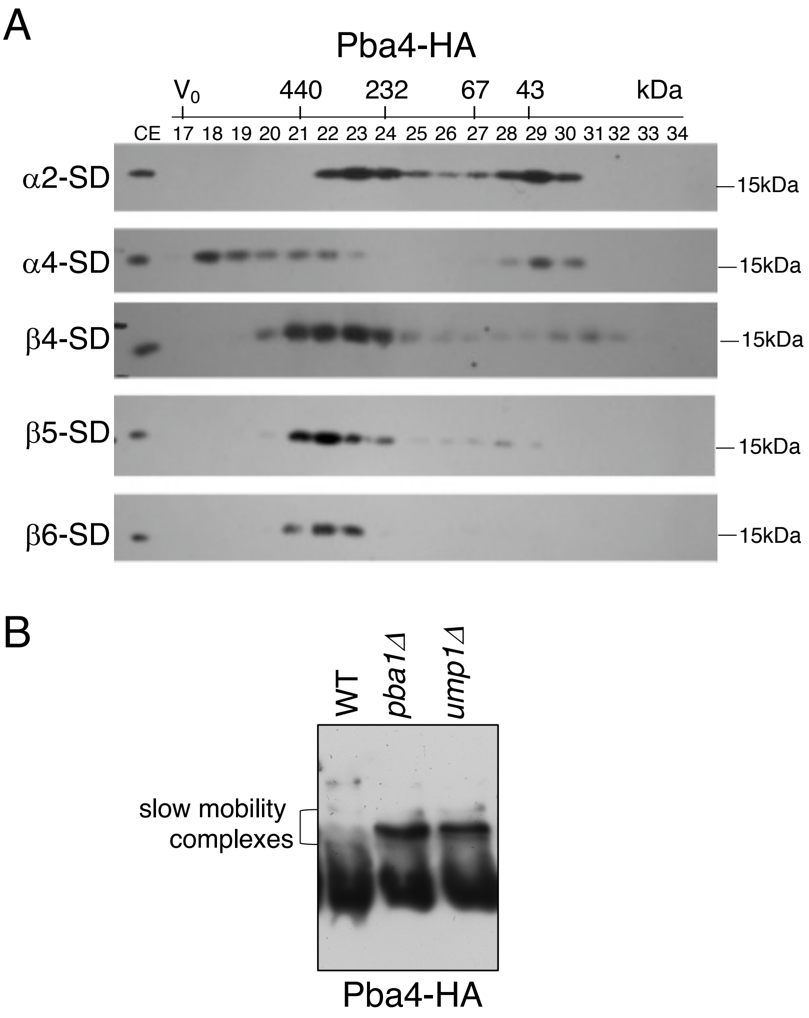

Figure S4. Effect of deletions and subunit shutdowns on Pba4-containing complexes. **(A)** Gel filtration profiles from Pba4-containing complexes when α4, β4, β5 or β6 were shutdown individually. Depletion from the α4 subunit causes shift in the complexes containing Pba4. **(B)** Native gel analysis of the effect of *um1pΔ* and *pba1Δ* on Pba4-containing complexes.

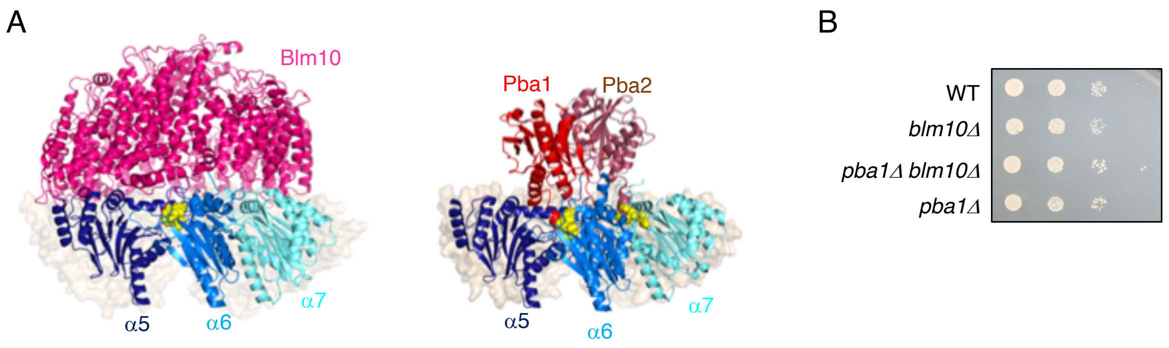

Figure S5. Alternative binding of Blm10 or Pba1-Pba2 to the CP α-ring surface. **(A)** Structural comparison of Blm10 and Pba1-Pba2 binding to the α-ring surface. Position of the HbYX motifs of Blm10, in the pocket between α5 and α6 subunit in the structure $^{pre1\text{-}1}$CP-Blm10 (PDB: 9GBK [18]), left, and of Pba1 and Pba2, between α5 and α6 subunit, and between α6 and α7 subunit respectively right (PDB: 8RVL [18]). HbYX residues are highlighted in yellow in spheres representation. **(B)** Deletion of *PBA1* and *BLM10* genes in the same cells do not affect growth in minimal media with glucose and causes cell advantage in media with galactose

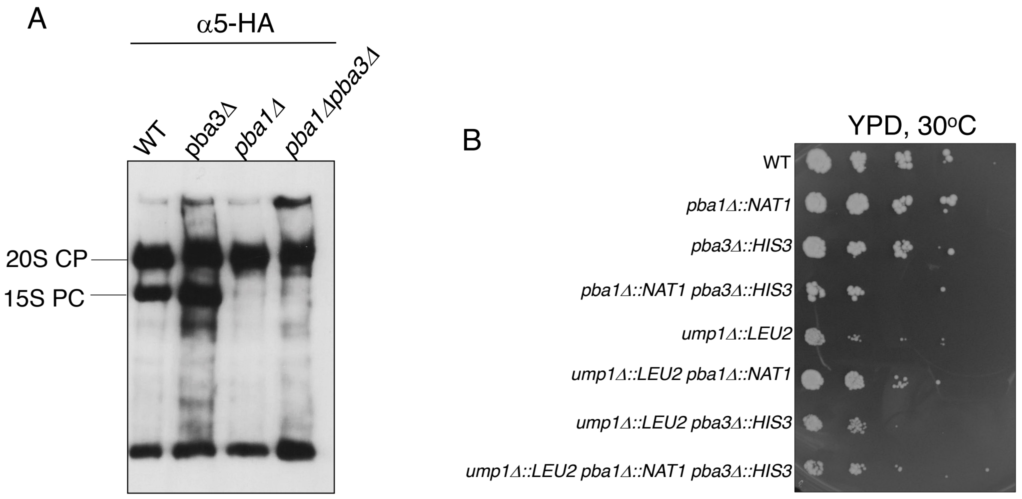

Figure S6. Pba chaperones are not essential for CP assembly. **(A)** α5 subunit-containing complexes from WT and different mutants were followed by native-PAGE, anti-HA blot. **(B)** Growth behavior of the indicated mutants in complete media.

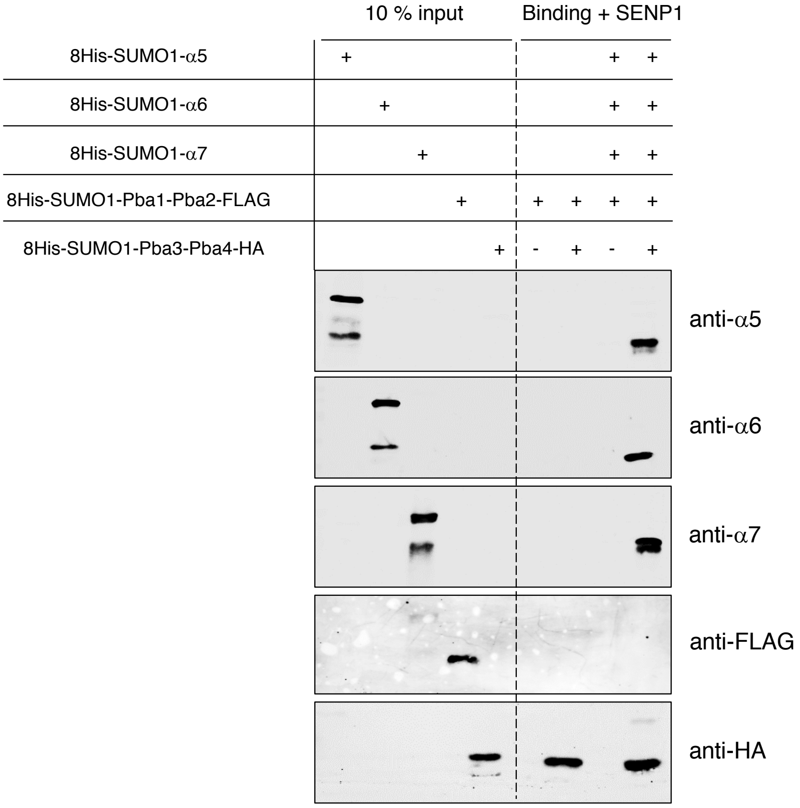

Figure S7. *In vitro* assembly of Pba3-Pba4 with α5, α6, and α7. Co-immunoprecipitation of Pba3-Pba4 with proteasome subunits α5, α6 and α7 and the chaperone pair Pba1- Pba2. Pba3-Pba4-HA was immobilized to HA resin and tested for binding to α5, α6, α7 and Pba1-Pba2-FLAG. All components were purified from *E. coli* as 8His-SUMO1 fusions, a solubility tag which was cleaved during the binding step. Bound proteins were eluted with imidazole and analyzed by SDS-PAGE and western blotting using specific antibodies (anti-α5, -α6, -α7, -FLAG and -HA). As a control, proteins were incubated without Pba3-Pba4-HA to exclude unspecific affinity to the resin. The two chaperone pairs Pba1-Pba2 and Pba3-Pba4 alone were tested for binding as well. For all utilized 8His-SUMO1 tagged proteins, 10 % input were loaded.

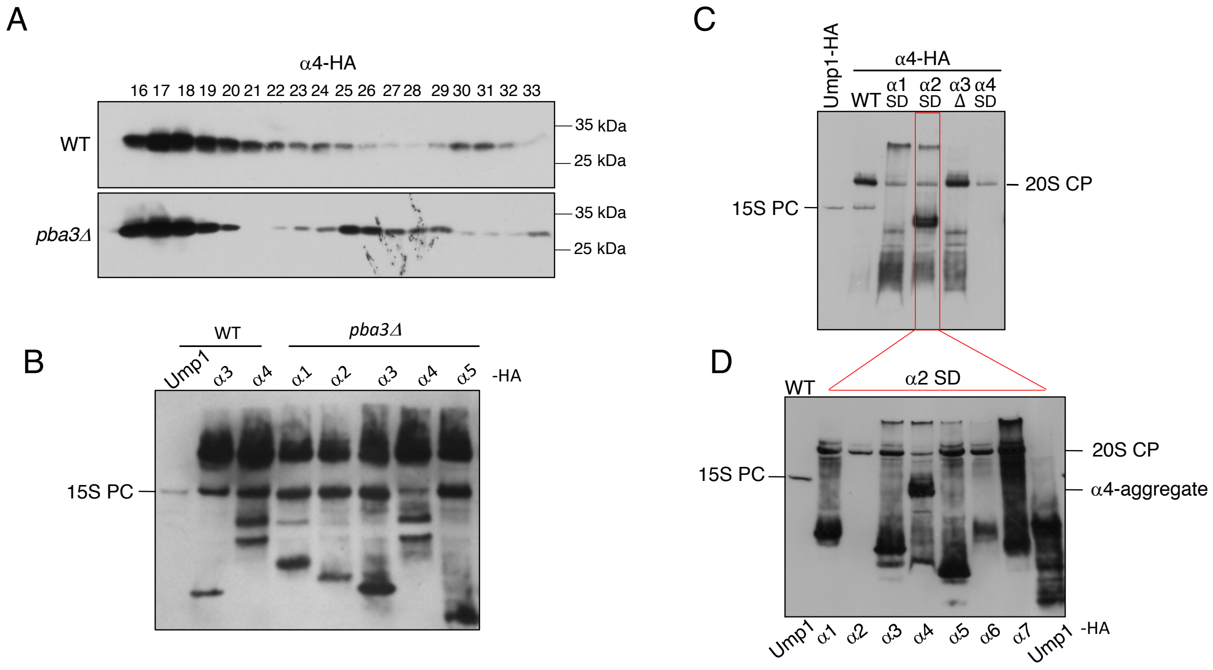

Figure S8. Analysis of α4 subunit-containing complexes in *pba3Δ* and several shut-down strains. (**A**) Crude extracts of the indicated strains were fractionated by Superdex 200 gel filtration. Fractions were analyzed by SDS-PAGE and anti-HA western blotting detecting Pba4-HA. (**B**) Comparison of mobilities of complexes containing the indicated HA-tagged α-subunits in wild type (WT) and *pba3Δ* cells by native gel analysis and anti-HA western blotting. Ump1-HA was loaded as a marker for 15S-PC. (**C**) α4 complexes in WT cells, or cells in which α1, α2, or α4 were shut-down or α3 deleted were analyzed by native PAGE and anti-HA western blotting. (**D**) Native PAGE analysis of extracts from cells after shutdown of α2/*PRE8* shutdown. Otherwise, identical strains were used, in which distinct α-subunits or Ump1 (as indicated) were tagged with HA.
