# Supplementary material for: The α5-α6-α7-Pba3-Pba4 Complex: A Starting Unit in Proteasome Core Particle Assembly
Source: Biomolecules. 2025 May 8;15(5):683. doi: 10.3390/biom15050683 (PMC12109424; doi:10.3390/biom15050683)

Matias et al. 2025

Uncropped Original Figures

Figure 1

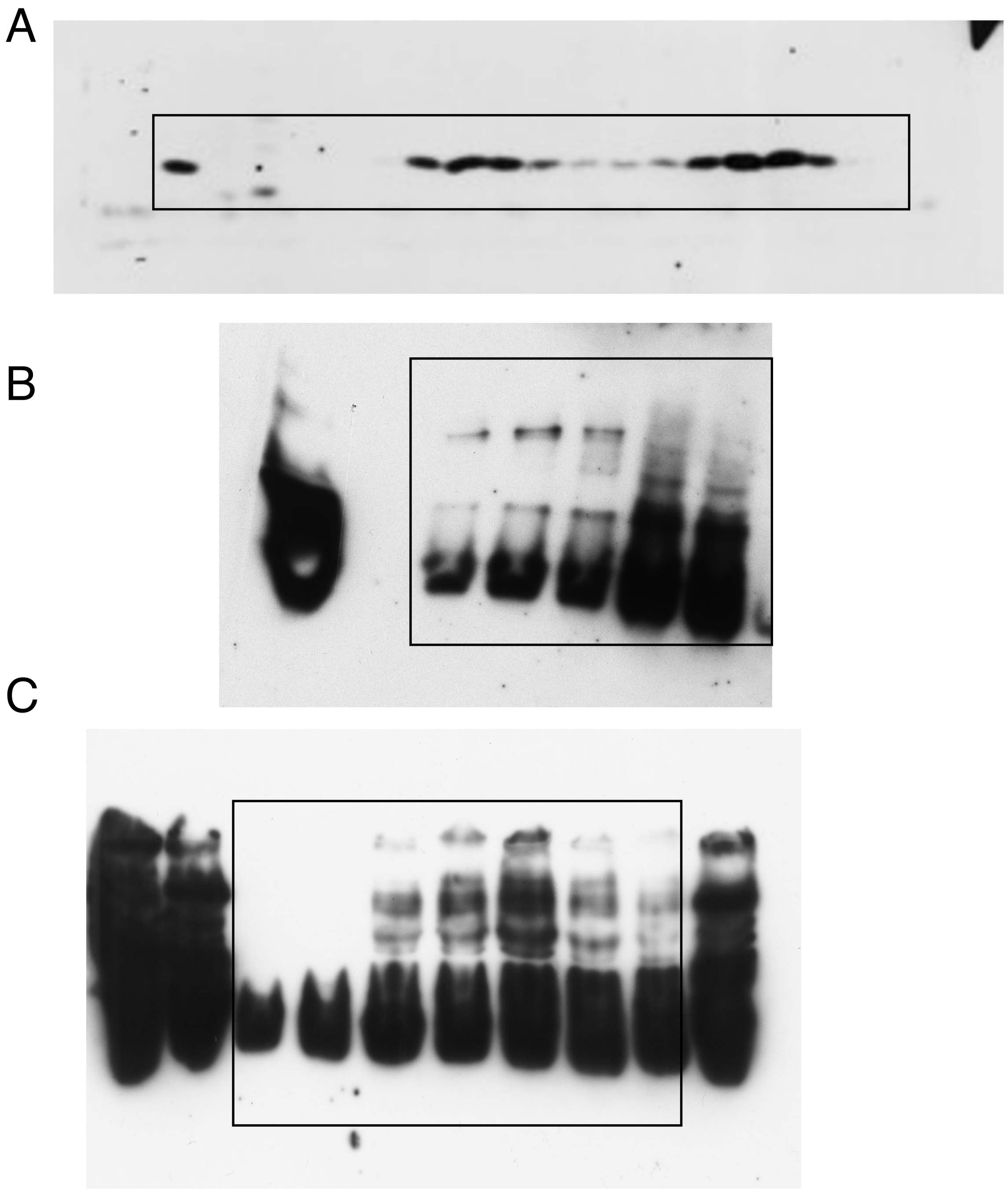

Figure 2

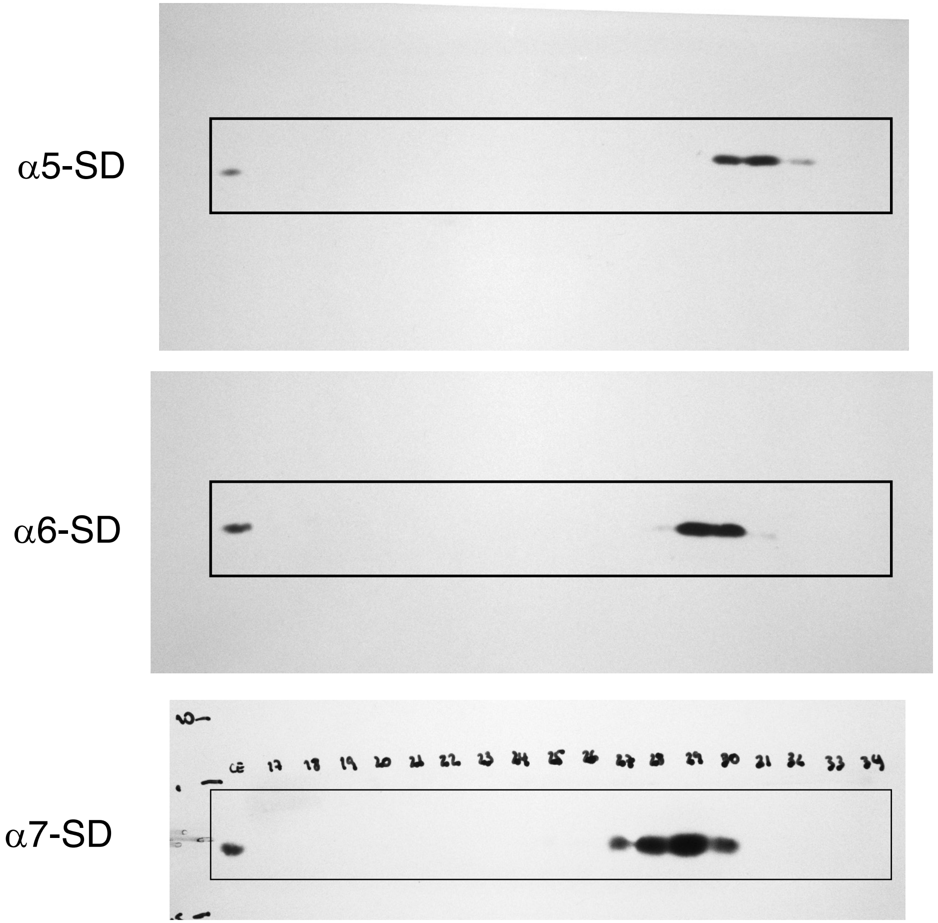

Figure 3

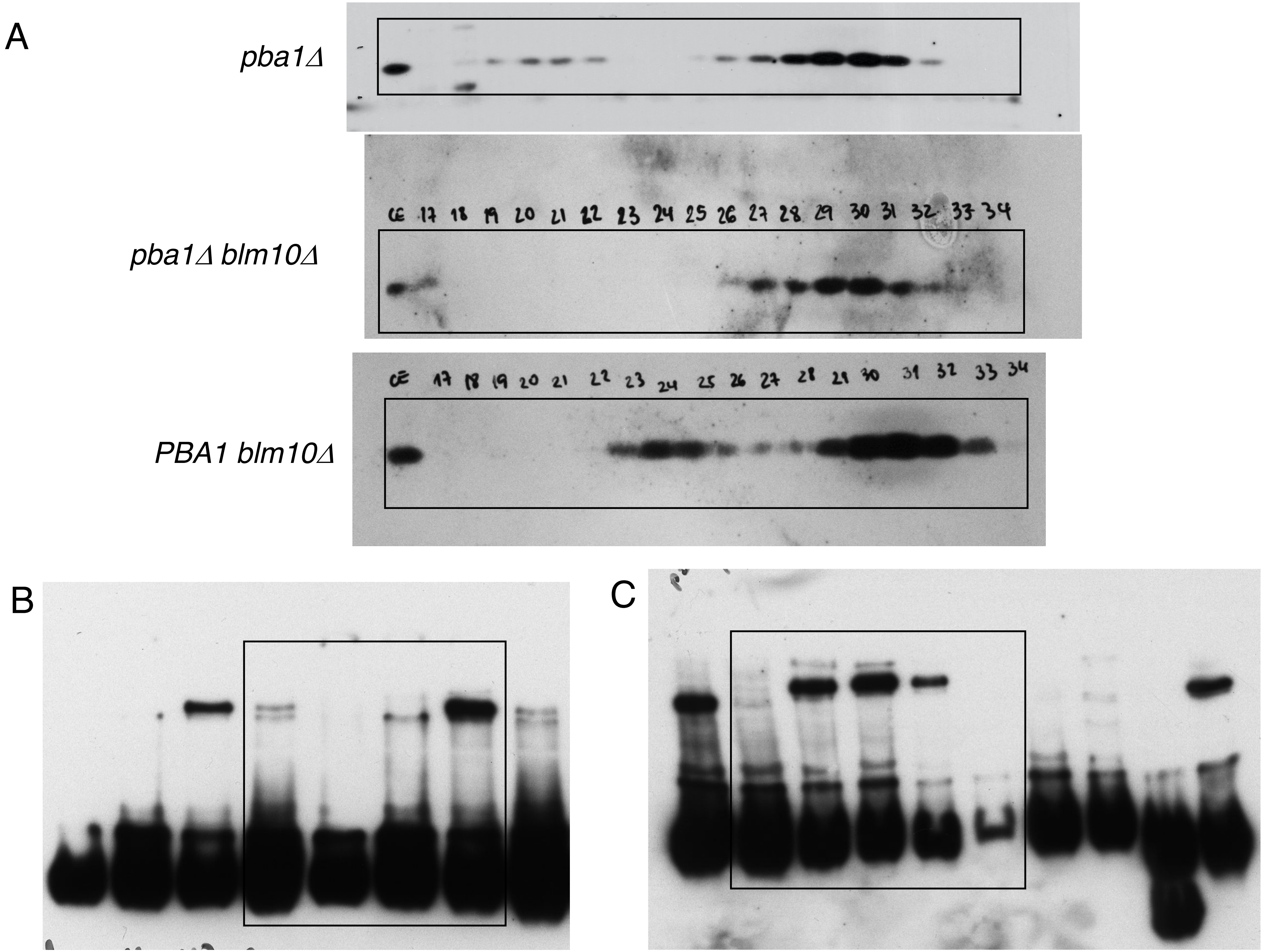
A
pba1Δ
pba1Δ blm10Δ
CE 17 18 19 20 21 22 23 24 25 26 27 28 29 30 31 32 33 34
PBA1 blm10Δ
CE 17 18 19 20 21 22 23 24 25 26 27 28 29 30 31 32 33 34
B
C

Figure 4

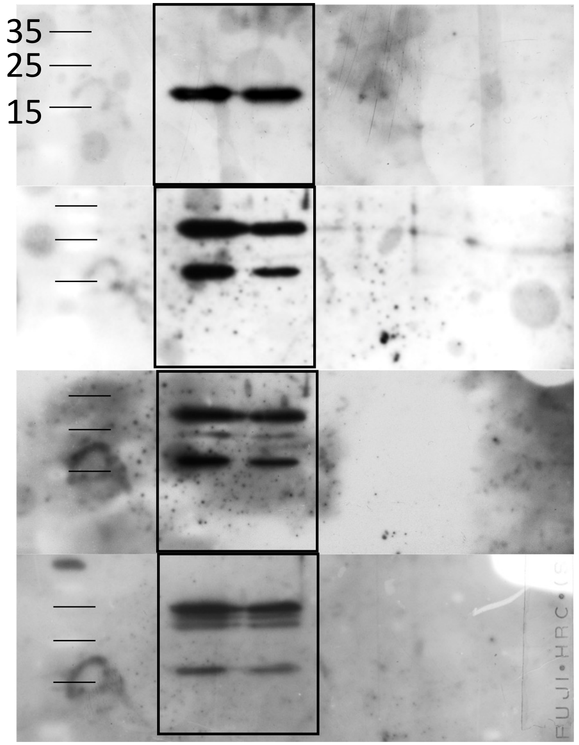

1st anti-FLAG

2nd anti-alpha5

3rd anti-alpha6

4th anti-alpha7

Figure 5

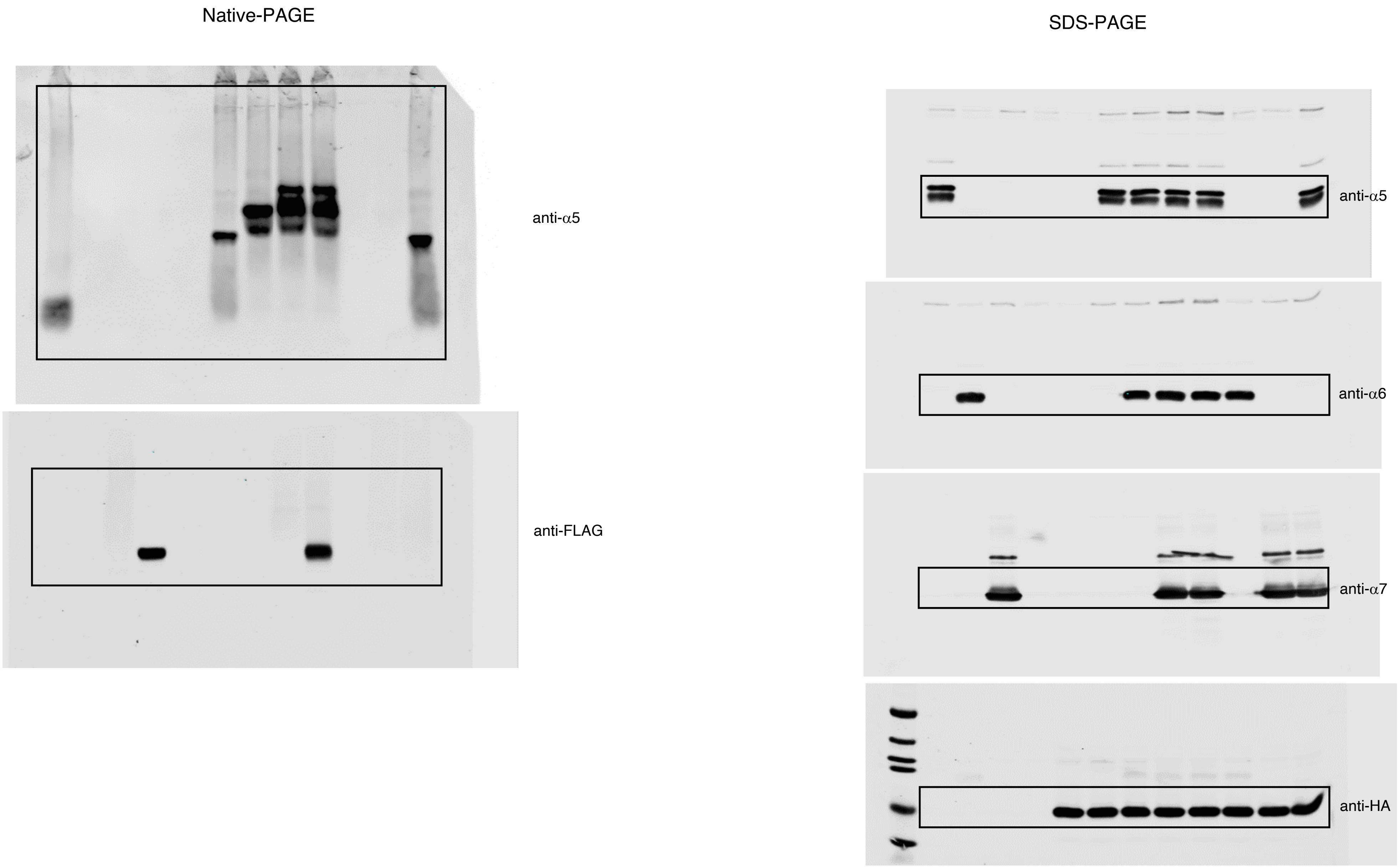
Native-PAGE
anti-α5
anti-FLAG
SDS-PAGE
anti-α5
anti-α6
anti-α7
anti-HA

Figure S1

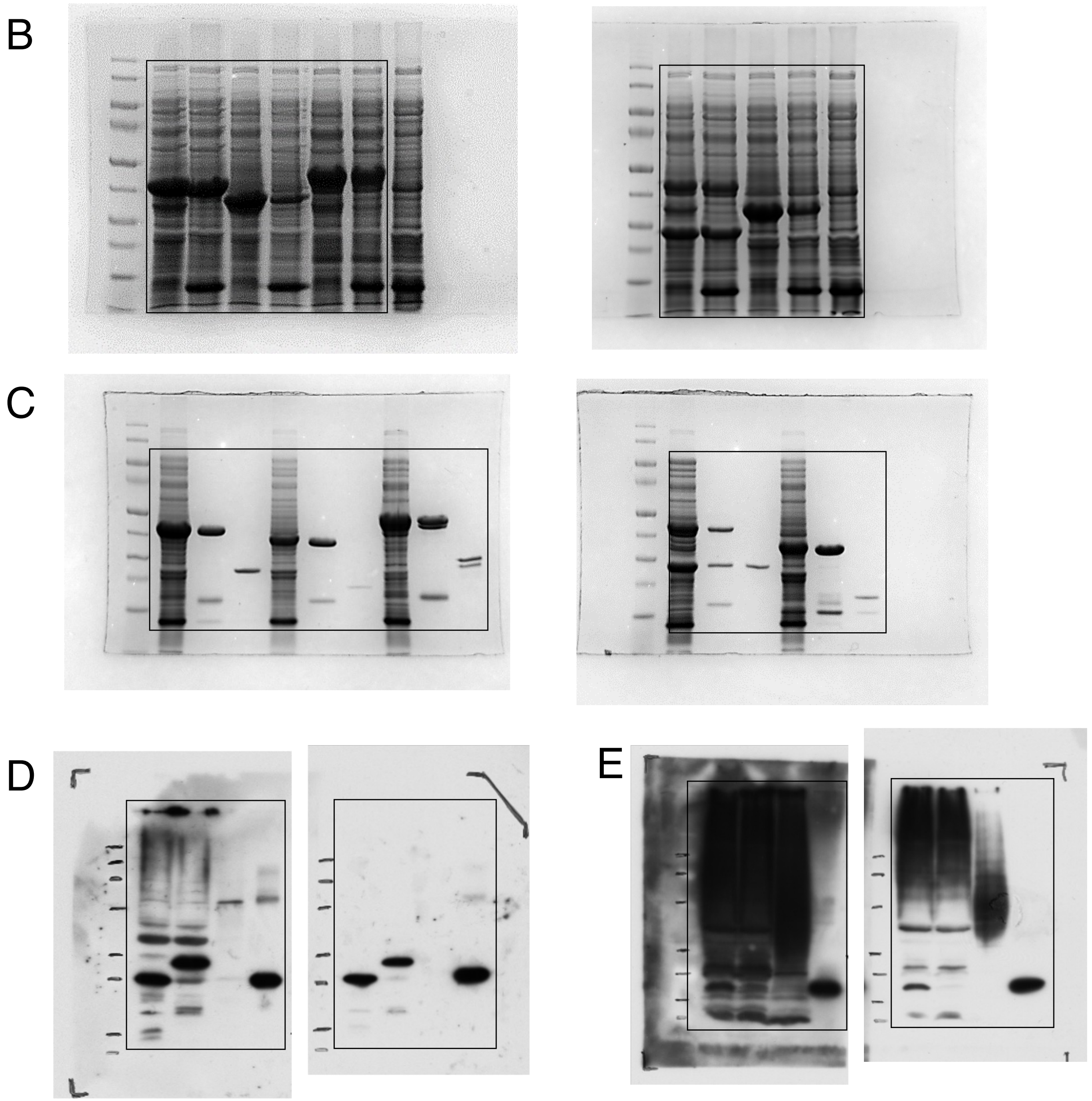

Figure S2

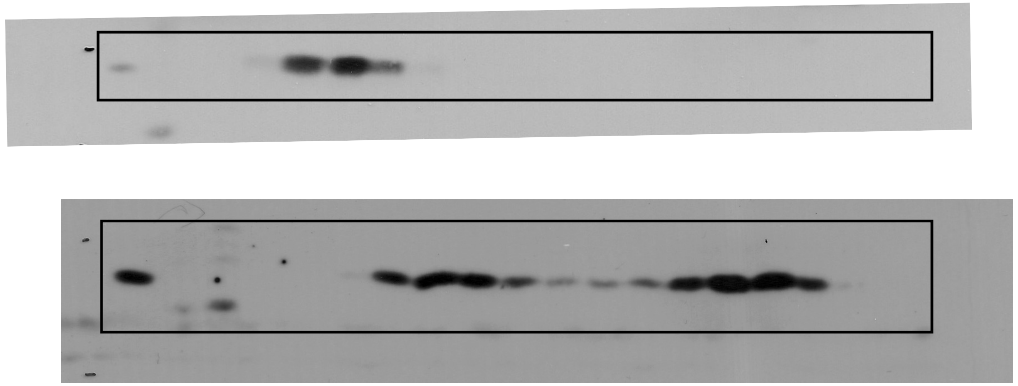

Figure S4

A

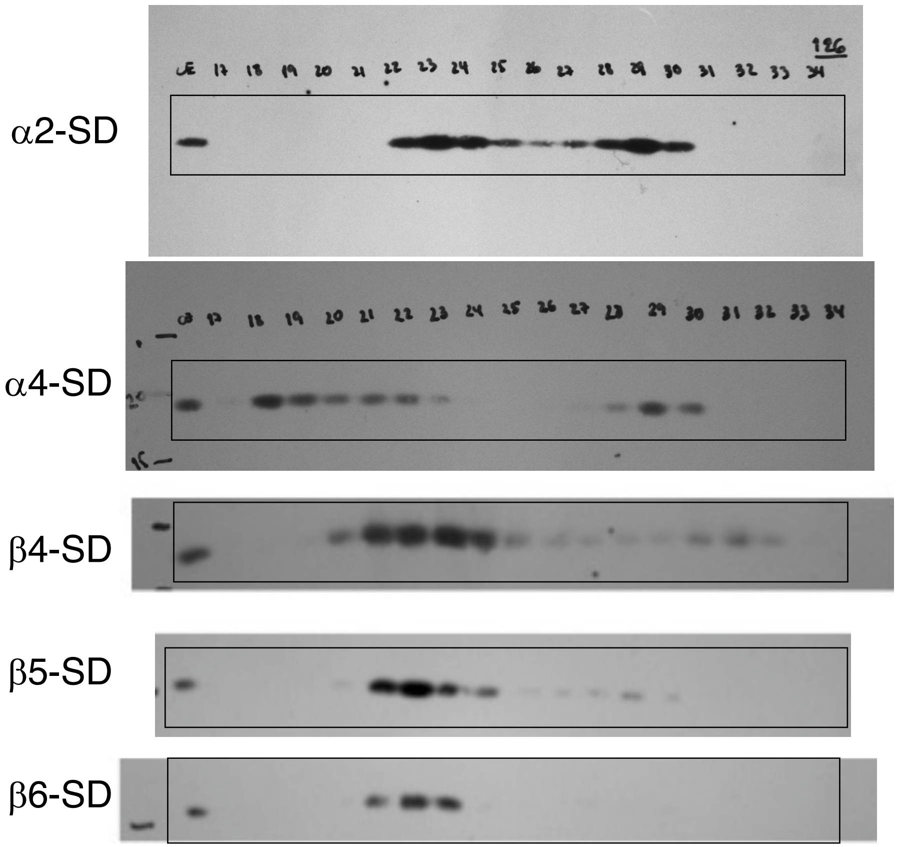

B

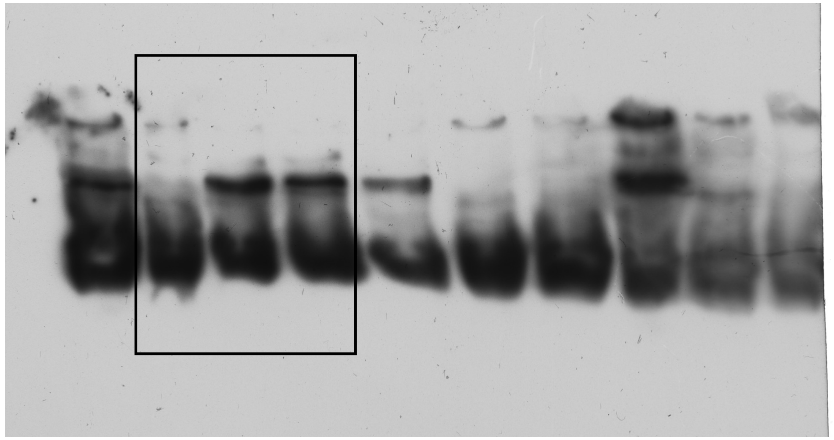

B

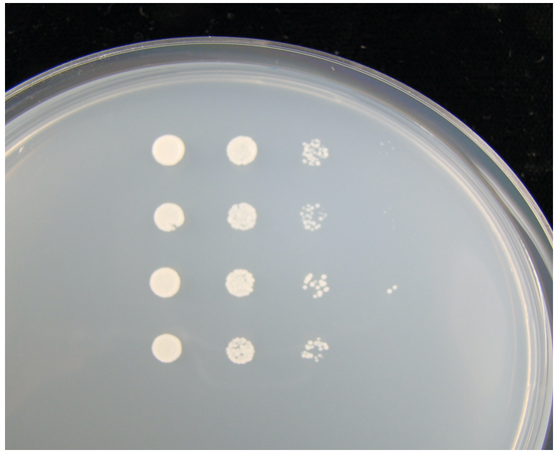

A

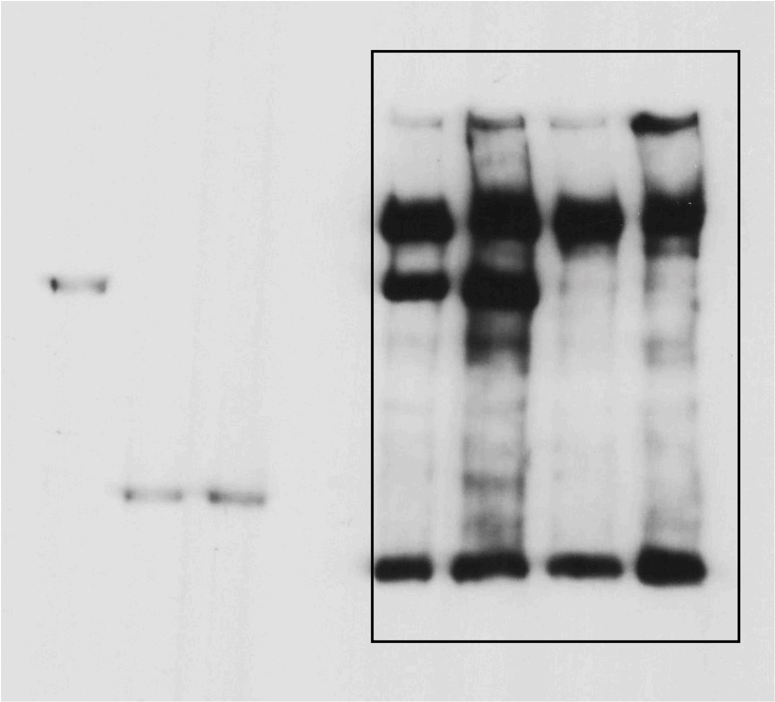

B

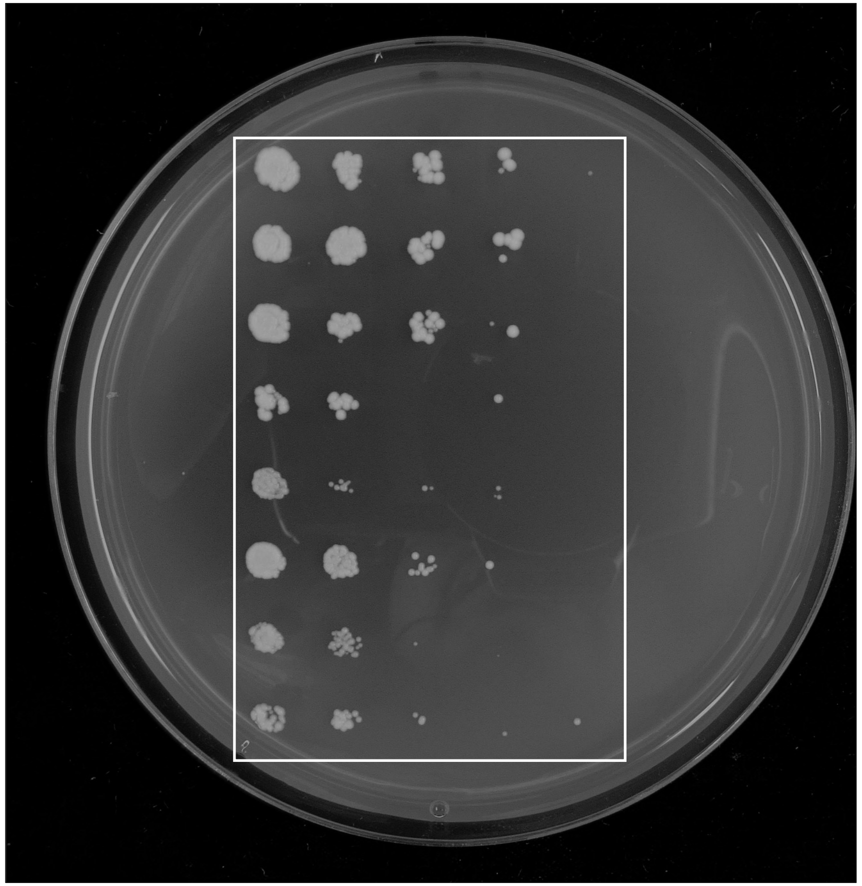

Figure S7

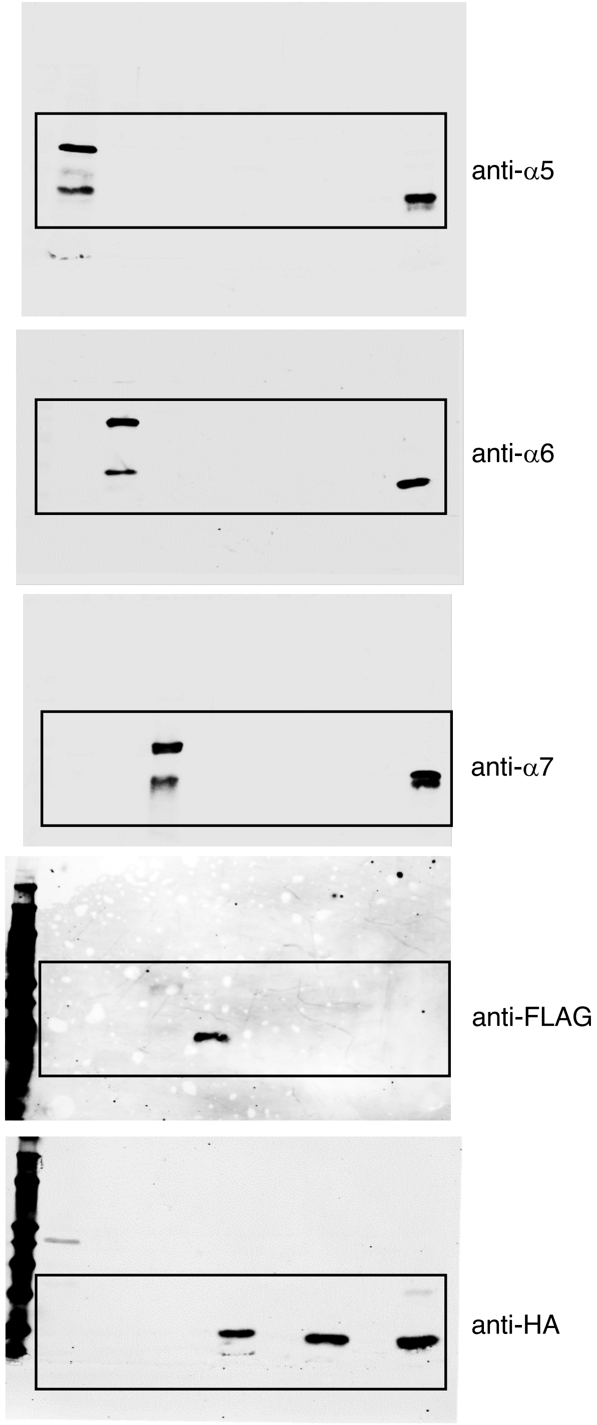

Figure S8

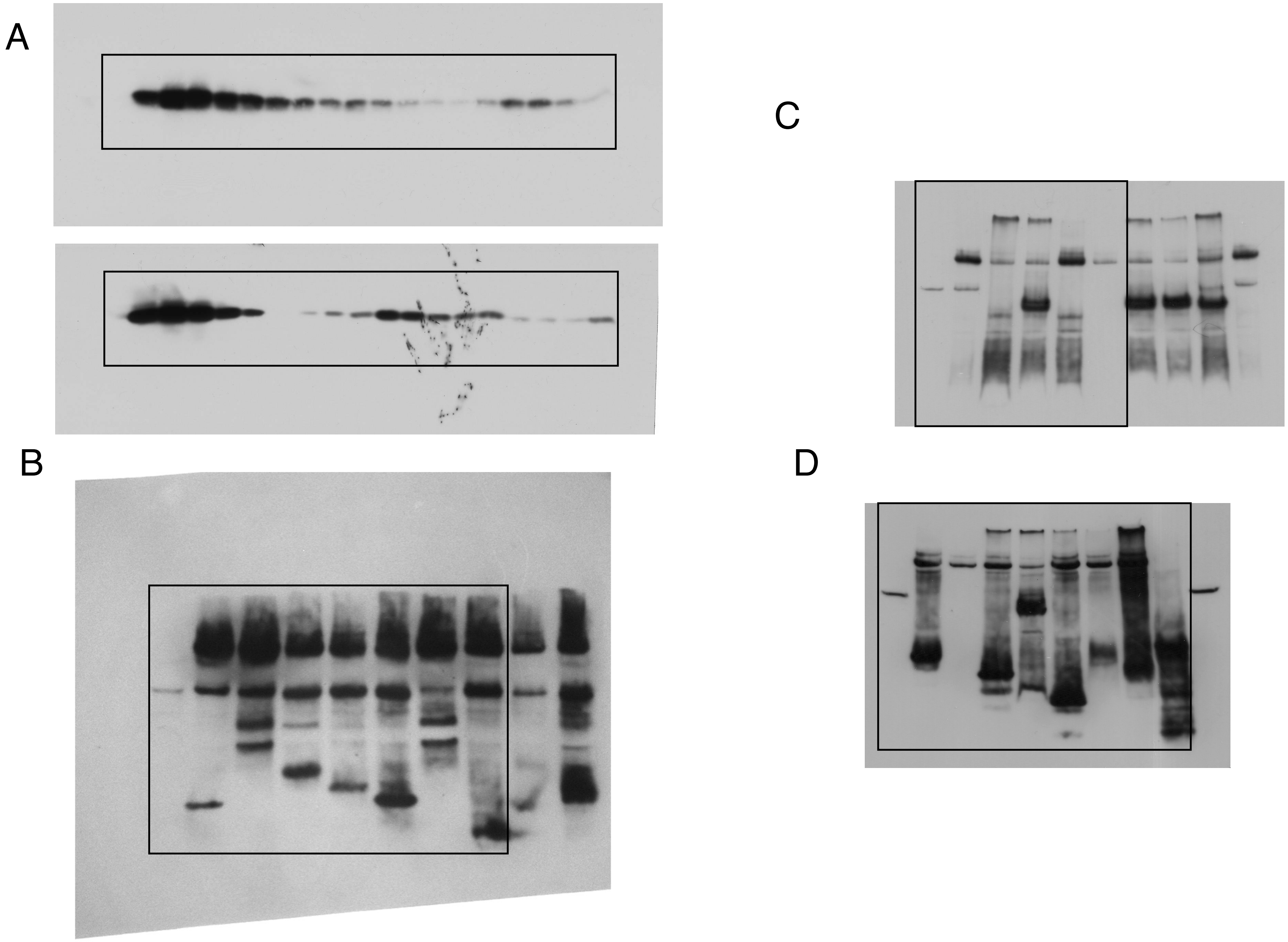

Supplement: Supplementary file 1 [file biomolecules-15-00683-s001.zip › biomolecules-3592808-Figure S9.pdf]
